# The impact of COVID-19 pandemic on tobacco use: A population-based study

**DOI:** 10.1371/journal.pone.0287375

**Published:** 2023-06-23

**Authors:** Mahmoud A. Alomari, Omar F. Khabour, Karem H. Alzoubi, Abubakar B. Maikano

**Affiliations:** 1 Division of Physical Therapy, Department of Rehabilitation Sciences, Jordan University of Science and Technology, Irbid, Jordan; 2 Department of Physical Education, Qatar University, Doha, Qatar; 3 Department of Medical Laboratory Sciences, Jordan University of Science and Technology, Irbid, Jordan; 4 Department of Pharmacy Practice and Pharmacotherapeutics, College of Pharmacy, University of Sharjah, Sharjah, UAE; 5 Department of Clinical Pharmacy, Jordan University of Science and Technology, Irbid, Jordan; 6 Department of Public Health and Disease Control, Kano State Ministry of Health, Kano, Nigeria; Aravind Eye Hospital and Post Graduate Institute of Ophthalmology, INDIA

## Abstract

**Background:**

Various aspects of lifestyle seem to change during confinement, particularly during the COVID-19 pandemic. The current study examines confinement’s effects on tobacco smoking habits (SH).

**Methods:**

A survey was distributed among adults living in Jordan (age >18 years) of both genders during April-May of 2020, of which 1925 responded to the survey.

**Results:**

The prevalence of smoking was 33.3%, 46.1%, and 21.1% for cigarettes (Cg), waterpipe (Wp), and E-cigarettes (ECg), respectively. Among the smokers, 38.5–45.8% reported a “no-change,” while 32.1–41.7% reported a “decrease” in SH during confinement. On the other hand, 18.0–22.1% reported an “increase” in the SH. However, concerning the factors that might affect SH, the results showed that age, gender, income, and job sector contribute to the observed changes.

**Conclusions:**

Changes in the SH during COVID-19 have been reported in about 50% of participants who smoke tobacco, with a more reported decrease than increase in use. Studies and interventions are needed to confirm further and understand the current results and discourage smoking during the COVID-19 pandemic.

## 1. Introduction

In December 2019, coronavirus (COVID-19) emerged with symptoms similar to respiratory syndrome 1 (SARS-CoV-1). In March 2020, the World Health Organization (WHO) declared the disease a pandemic. Since then, the disease has spread worldwide, resulting in millions of fatalities as of June 2022. According to the WHO, many control strategies have been implemented worldwide to curb the COVID-19 epidemic. Public health strategies include enlightenment, decontamination, isolation, and contact tracing [1, 2]. Apart from public health containment strategies, various mitigation measures were implemented by different countries and regions [3]. For instance, China has effectively adopted quarantine and social distancing according to the WHO-COVID-19 situation report 44. Restriction of movements has reportedly delayed the onset of outbreaks in other areas of Wuhan [4], while national lockdowns were imposed in some countries, such as India [5, 6] and Germany [7]. Adopting such measures has been shown to impact the different lifestyles of people, including sleeping habits, food intake, exercise, and others [8–10].

Smoking is a lifestyle component that refers to the intake of smoke from burning tobacco into the lungs orally. The most common form of tobacco products are cigarettes (Cg), waterpipe (Wp), and recently heated tobacco products [11, 12]. In addition, electronic nicotine delivery systems, also known as electronic cigarettes (ECg), are becoming popular all over the globe [13]. Smoking is a known health hazard as it exposes the body to numerous toxic compounds that cause diseases, including respiratory illnesses and lung cancer [14]. The most harmful smoke toxicants are nicotine, carbon monoxide, and carcinogens like tobacco-specific nitrosamines [15]. These compounds are pro-inflammatory, suppressive to the immune system, and negatively interfere with the normal tissue repair process and host response to foreign antigens [16–18]. Smoking has been ranked among the leading causes of diseases, hospitalization, morbidity, and mortality worldwide [19]. Smoking also has been implicated in the risk and etiology of cardiovascular, respiratory, immune, and metabolic diseases [20]. Recent literature has shown that smoking is associated with negative progression and adverse outcomes of COVID-19 [21, 22]. Despite these adverse health effects, many smokers claim that smoking helps with psychological stress, including boredom, anxiety, and depression commonly associated with compulsory confinement [23]. Accordingly, isolation is associated with a greater risk of smoking. For example, a study among university students in Tehran showed that social isolation is more prevalent among smokers [24]. Cg smoking was also higher in prisoners [25] and healthcare personnel in humanitarian missions [26]. During the COVID-19 pandemic, some studies recommended quitting smoking as a preventative measure for infection because tobacco consumption adversely affects the immune system [27]. In addition, being a current smoker is associated with higher stress scores [28].

Few studies have investigated the impact of disease-induced confinement (such as quarantine, isolation, and lockdown) as applied in the context of COVID-19 on SH. For example, a qualitative study of 25 adult smokers reported increased smoking due to the COVID-19 lockdown [29]. A study of university students reported a higher risk of substance use and smoking during the pandemic lockdown [30]. Changes in smoking habits have been reported by some studies conducted during the COVID-19 pandemic triggered mainly by smokers’ beliefs regarding the risk of virus transmission/magnitude of the symptoms imposed by smoking [31, 32]. This study seeks to investigate the effects of confinement on SH in Jordan, a country with one of the highest prevalence rates of tobacco use globally [33–35]. The study will specifically determine the increase, decrease, or no change in smoking Cg, Wp, and ECg. In addition, factors associated with changes in SH were investigated. The findings of this study will contribute to recognizing the direction of change in SH during disease-induced confinement. Subsequently, contemplate the potential benefits of confinement in promoting positive change in SH. Moreover, the findings might help plan and implement tactics and strategies to mitigate the negative health effects of smoking and restrain the potential tobacco spread during current and future calamities.

## 2. Method

### 2.1. Design and participants

The data for the current study was obtained from the “Behavior, Knowledge, Stress and Quality of Life during COVID-19-induced Confinement (BKSQ-COVID-19) project” [8]. The study is cross-sectional to examine changes in SH. The study survey was created on Google Forms and was distributed among Arabic-speaking adults (age >18 years) in Jordan of both genders during April-May 2020. Excluded were those who did not read Arabic or had no access to social media platforms. Social media platforms (Facebook, WhatsApp., etc..) were used to anonymously and electronically distribute the questionnaire. The researcher used G*Power software version 3.1.9.7 to calculate the sample size. A 0.05 significance level, a power of 0.90, and a small effect size of 0.10 required the minimum number of subjects to be 1810. A snowball sampling design was used where each study participant was asked to nominate others from their social network until the desired number of participants was achieved. The study’s research team followed up on participants’ recruitment to ensure proper representation of most settings, spectra, and entities in Jordanian society. The study survey was in Arabic, as those who do not speak Arabic are less than 0.5% of the population in Jordan.

### 2.2. Ethics approval and consent to participate

The study was approved by the institutional review board at the Jordan University of Science and Technology, Irbid-Jordan (245/2020). Before completing the questionnaire, the participants were informed about the study’s objectives and consented electronically.

### 2.3. Questionnaire

The research team developed the questionnaire for the current study based on similar studies [36]. Information about demographics, socioeconomics, perceptions about COVID-19 disease, and changes in SH during the pandemic was obtained. The participants were asked to self-report age, gender, weight, height, job sector (i.e., government and private), education, and income. The income contained three categories: low (less than 750 JD), medium (750-less than 1500 JD), and high (more than 1500 JD), which is in accordance with the Jordan Department of Statistics classification of income in Jordan. Additionally, the participants were asked about the likelihood of getting infected, knowing somebody infected with COVID-19, and the implemented governmental confinement procedures. The response options were either “Yes” or “No”. A “Yes” response indicates that the participant was subjected to the confinement procedure. At the same time, a “No’ answer means that the participant was not subjected to the confinement procedures. The survey asked the participants about changes in three types of smoking: Cg, Wp, and ECg. The questions were: “What changes have you experienced in the following smoking types due to confinement imposed by COVID-19? Three choices were available, “increase”, “decrease”, and “no change”. The participants were asked to report the changes in frequency and amount of all types of smoking at the time of data collection. The responses to the demographics, socioeconomics, perceptions about COVID-19 disease, and changes in SH during the pandemic were obtained according to the participants’ self-perception.

The initial draft of the survey was content and face-validated to a group of experts to provide their feedback, and the survey was modified accordingly. Then, the final draft of the questionnaire was pilot tested with a group of 50 participants to offer their advice regarding the clarity and comprehensibility of the questions. Finally, the responses from those participants were excluded from the final analysis. Finally, the reliability of each questionnaire’s items was calculated, ensuring that Cronbach’s alpha was more than 0.65.

### 2.4. Statistics

The data was entered and coded in the SPSS (version 21) for statistical analysis and presented as mean±SD, frequency, and percentages. Significance (*p*-value) was set at 0.05. To examine the differences in the participant responses to the smoking questions, the **χ**^**2**^ goodness-of-fit was used among the subset of the study sample that were current smokers (n = 851). Additionally, multinomial logistic regression was used to determine the relationship of the potential factors with the participant responses to the questions. The potential factors were age, gender, obesity, income, education, and job sector.

## 3. Results

### 3.1. Participants

The data was collected from 1925 individuals, of which 79 (4.1%) reported no smoking data and were excluded from the analysis. Thus, the remaining 1844 participants were included in the analysis. As shown in Table 1, the participant age, weight, and height ranges were 18–72 years, 38–144 kg, and 120–198 cm, respectively. A greater percentage of the participants were women, with a bachelor’s degree, receiving middle income, and work in the governmental sector. In Table 2, the confinement tactics implemented in Jordan are reported. These tactics include self-quarantine, social distancing, lockdown, school closure, and event banning reported by most participants.

**Table 1:** The participant demographic (n = 1844).

Gender (%; male)		30.5
Age (yrs, mean±SD)		33.7 ± 11.3
Weight (kg, mean±SD)		72.6± 16.3
Height (cm, mean±SD)		166.3± 9.0
Level of Education (%)		
	High school and less	19.4
	Community college diploma	14.1
	Bachelor’s degree	51.3
	Postgraduate degree	15.3
Income (%)		
	Low	16.2
	Middle	76.0
	High	7.8
Job sector (%)		
	Government	50.7
	Private	23.8
	Unemployed/retired	24.3

**Table 2:** Confinement information due to COVID19 (n = 1844).

Likelihood of getting infected		(%)
	Low	59.5
	Moderate	34.5
	High	6.0
Know somebody who is infected		
	Yes	6.3
	No	93.7
Self-quarantine		
	Yes	93.5
	No	6.5
Physical distancing		
	Yes	96.8
	No	3.2
Banning group events (i.e. weddings)		
	Yes	98.2
	No	1.8
School closure		
	Yes	99.0
	No	1.0
Lockdown		
	Yes	97.0
	No	3.0

Confinement tactics during the COVID-19 pandemic in Jordan

### 3.2. Prevalence of smoking

As depicted in Table 3, the majority (53.9–78.9%) of the participants reported no smoking of any kind (Cg, Wp, or ECg), with Wp being the most prevalent (46.1%) of the sample.

**Table 3:** Prevalence of smoking during COVID19 (n = 1844).

Type of Smoking	Smokers	Nonsmokers
Cigarettes (%)	33.3	67.7
Waterpipe (%)	46.1	53.9
E-cigarettes (%)	21.1	78.9

Values presented are the percent (%) of participants

### 3.3. Changes in smoking habits

The chi-square goodness-of-fit test demonstrates differences (*p*<0.0001) in the responses to the SH questions, “increase”, “decrease”, versus “no-change”. Table 4 shows that among the smokers, 38.5–45.8% reported a “no-change” and 32.1–41.7% reported a “decrease”, while about 20% (range: 18.0–22.1) reported an “increase” in the SH during confinement.

**Table 4:** Prevalence of changes in smoking habit during COVID19 (n = 851).

Smoking habits	Decreased	No change	Increased	χ^2^; *p*-value
Cigarettes (%)	32.1	45.8	22.1	50.9; 0001
Waterpipe (%)	41.7	38.5	19.7	72.0; 0001
E-cigarettes (%)	39.1	41.9	18.0	37.5: 0001

Values presented are the percent (%) of participants

### 3.4. Factors contributing to the changes in smoking habits

The regression model explained 14.2–16.4% (χ^2^ = 256.6; *p*<0.0001) of the variation in smoking Cg. Further analysis showed that age (χ^2^ = 12.1; *p*<0.007), gender (χ^2^ = 167.3; *p*<0.0001), and education (χ^2^ = 26.1; *p*<0.002), but not obesity (χ^2^ = 5.7; *p*>0.12), income (χ^2^ = 8.6; *p*>0.20), or job sector (χ^2^ = 4.1; *p*>0.667), were related to smoking Cg.

Additional regression analysis explained 8.0–8.8% (χ^2^ = 140.4; *p*<0.0001) of the variation in smoking Wp. Subsequent analysis showed that only age (χ^2^ = 16.9; *p*<0.001), gender (χ^2^ = 47.7; *p*<0.0001), education (χ^2^ = 35.2; *p*<0.0001) and income (χ^2^ = 15.7; *p*<0.016) were related to smoking Wp, but not obesity (χ^2^ = 2.4; *p*>0.50) or job sector (χ^2^ = 4.7; *p*>0.557), were related to smoking Cg.

Another regression analysis explained 5.6–7.4% (χ^2^ = 96.8; *p*<0.0001) of the variation in smoking ECg. Further analysis showed that only gender (χ^2^ = 39.0; *p*<0.0001) and income (χ^2^ = 22.4; *p*<0.0001), but not age (χ^2^ = 6.6; *p*>0.08), obesity (χ^2^ = 1.4; *p*>0.75), education (χ^2^ = 8.9; *p*>0.440), or job sector (χ^2^ = 11.9; *p*>0.06), were related to smoking Cg. See S1 Appendix for the details of the regression results.

## 4. Discussions

In compliance with WHO recommendations, governments across the globe have imposed a variety of confinement measures to overcome the COVID-19 pandemic [37]. The current study was designed to determine the changes in SH during confinement due to COVID19. Additionally, the factors contributing to these changes were also examined.

A variety of restriction measures, including self-quarantine, social distancing, banning of group events, school closure, and lockdowns, affected most (93.7–99.0%) of the study participants, with the majority (53.9–78.9%) reporting no smoking of any type. Across the smoking categories, the majority reported either “no-change” (38.5–45.8%) or “decrease” (32.1–41.7%) in smoking during the confinement. Additionally, changes in Cg smoking were found to be associated with age and obesity.

The majority of participants in the current study reported a no-change or a decrease in SH during the imposed restrictions, which is in line with the findings of similar studies from Arab countries [38] and Italy [10, 39]. In Saudi Arabia, employment smoking decreased significantly during the pandemic [38]. Additionally, up to 46% of the participants reported no change in lifestyle, including smoking patterns in Italy [10, 39]. However, a significant proportion (13%) demonstrated a slight decrease in the use of tobacco products. The decrease might be due to denied opportunity to live the usual social life [40], the influence of the non-smoking cohabiting partners [41], concern about exuberating COVID-19 symptoms [21] and fear of contracting COVID-19 [42] or dying from it following infection [43]. Lack of access to tobacco products due to restriction of movement, closure of shops, and circulation of cash may also be contributing factors.

In contrast, previous studies from Jordan and neighboring countries showed that more participants reported an increase versus a decrease in smoking [31, 34, 44]. For example, in a study from Jordan, about 28% and 19% of tobacco users reported an increase or decrease in smoking during the pandemic, respectively [31]. In a study from Saudi Arabia, about 12% of the respondents reported increased tobacco use during the pandemic lockdown [44]. Similarly, Koreans reported increased smoking attributed to confinement-related stress, while the decrease was due to health-related conditions [45, 46]. Confinement-related stress has been reported to promote SH and influence its initiation. Accordingly, up to 9% and 10% of smoking initiation among blacks and non-blacks, respectively, was related to stress during incarceration [47]. As a strategy to counter the negative influence of detention, smoking cessation programs were considered essential elements during confinement. Within this realm, counseling and cessation therapy was effective in tapering down smoking among some detainees (>30% of study participants) [48].

The current results showed that changes in Cg and Wp smoking are associated with age, gender, income, and education, whereas changes in ECg smoking were related to gender and income.

According to data from several Arab countries, age, gender, education level, country of residence, and work status contributed to smoking behavior during the pandemic [49]. Similarly, a previous study [50] has attributed the changes in smoking to demographics (i.e., age and gender) and socioeconomic (i.e. education and income) factors. For instance, according to the US National Health Interview Survey report, individuals aged ≥25 with 9–11 years of education are more likely to be current, ever, and heavy smokers than those with 0–8 years [51]. However, with more than 11 years of education, the tendency to quit smoking was associated proportionally with an increase in education. It is important to mention that confinement is associated with mobility restrictions thus, limited accessibility to currency, goods, and services [52]. Hence, many cigarette smokers most likely might be denied access to tobacco products during the COVID-19 disease, thus experiencing a decrease in tobacco smoking. However, future studies are needed to confirm the current factors and to verify these speculations.

A decrease in SH is likely due to COVID-19 confinement reported in about one-third of the sample, which may be attributed to the inaccessibility of tobacco products, restraints in social life, the influence of living partners, or fear of getting the disease. Accordingly, an opportunity at sight for promoting positive smoking behavior during COVID-19 induced confinement and other similar situations. This could be achieved by developing policies and implementing strategies such as effective awareness and motivational campaigns.

## 5. Implications

According to the results, the majority of the participants reported a decrease or no-change in smoking COVID19 confinement. This may be attributed to inaccessibility to tobacco products due to restraints in social life, the influence of living partners, immobility, and fear of getting the disease. Accordingly, an opportunity at sight for promoting positive smoking behavior during COVID19-induced confinement; therefore, strategies are needed to implement policies encouraging positive behaviors, including smoking cessation programs. These strategies might include the development of effective awareness and motivational campaigns.

## 6. Limitations

The study was a cross-sectional and conducted on a relatively small sample size in a fairly small Middle-Eastern country (i.e. Jordan). Thus cause-effect inferences and the generalizability of the results are limited. Additionally, other factors that might contribute to changes in smoking behavior and the possible effects of these changes are not examined in the current study. Therefore, multi-country longitudinal studies with a larger sample are needed. Additionally, future studies should be mechanistic and include more potential factors that might contribute to changes in smoking behavior.

## 7. Conclusions

Among the smokers, a low percentage reported increases in smoking during COVID19-induced confinement, while the majority experienced no change or decrease. Furthermore, the data shows that age, gender, income, and job sector contribute to these changes. However, studies and interventions are needed to confirm and understand the current results. Additionally, programs and strategies are required to discourage smoking during current and future similar calamities.

## Supporting information

S1 Appendix Factors contributing to changes in smoking habits during COVID19. (DOCX)

S1 Data (XLSX)
