# Supplementary material for: The impact of COVID-19 pandemic on tobacco use: A population-based study
Source: PLoS One. 2023 Jun 23;18(6):e0287375. doi: 10.1371/journal.pone.0287375 (PMC10289305; doi:10.1371/journal.pone.0287375)
Supplement: S1 Appendix — (DOCX) [file pone.0287375.s001.docx]

| **Appendix 1:** Factors contributing to changes in smoking habits during COVID19 | | | | | |
| --- | --- | --- | --- | --- | --- |
| **Cigarettes Smoking** | **Response: Increase** | | | | |
|  | **Factors** | **Level** | **P-value** | **OR** | **95% CI** |
|  | **Gender** | Men | 0.250 | 1.3 | 0.82-2.11 |
|  |  | Women | Reference | | |
|  | **Age** | ------------------------------------- | 0.003 | 0.97 | 0.95-0.99 |
|  | **Education** | High school and less | 0.639 | 1.219 | 0.53-2.785 |
|  |  | Community college diploma | 0.818 | 1.110 | 0.456-2.701 |
|  |  | Bachelor’s degree | 0.610 | 1.213 | 0.578-2.545 |
|  |  | Postgraduate degree | Reference | | |
|  | **Obesity** | ------------------------------------- | 0.712 | 0.991 | 0.942-1.041 |
|  | **Income** | Low | 0.067 | 2.776 | 0.932-8.269 |
|  |  | Middle | 0.065 | 2.571 | 0.942-7.011 |
|  |  | High | Reference | | |
|  | **Job Sector** | Government | 0.245 | 1.403 | 0.793-2.485 |
|  |  | Private | 0.087 | 1.676 | 0.928-3.029 |
|  |  | Unemployed/retired | Reference | | |
|  | **Response: Decrease** | | | | |
|  | **Gender** | Male | 0.677 | 0.914 | 0.600-1.394 |
|  |  | Female | Reference | | |
|  | **Age** | ------------------------------------- | 0.137 | 0.986 | 0.969-1.004 |
|  | **Education** | High school and less | 0.861 | 1.063 | 0.538-2.099 |
|  |  | Community college diploma | 0.326 | 0.684 | 0.320-1.460 |
|  |  | Bachelor’s degree | 0.315 | 0.734 | 0.402-1.341 |
|  |  | Postgraduate degree | Reference | | |
|  | **Obesity** | ------------------------------------- | 0.278 | 1.024 | 0.981-1.068 |
|  | **Income** | Low | 0.686 | 1.192 | 0.509-2.791 |
|  |  | Middle | 0.244 | 1.548 | 0.743-3.226 |
|  |  | High | Reference | | |
|  | **Job Sector** | Government | 0.669 | 1.112 | 0.682-1.813 |
|  |  | Private | 0.858 | 0.954 | 0.567-1.604 |
|  |  | Unemployed/retired | Reference | | |
|  | **Response: No change** | | | | |
|  | **Gender** | Male | 0.000 | 0.215 | 0.157-0.295 |
|  |  | Female | Reference | | |
|  | **Age** | ------------------------------------- | 0.005 | 0.981 | 0.967-0.994 |
|  | **Education** | High school and less | 0.001 | 0.415 | 0.246-0.703 |
|  |  | Community college diploma | 0.017 | 0.513 | 0.296-0.888 |
|  |  | Bachelor’s degree | 0.006 | 0.536 | 0 .344-0.835 |
|  |  | Postgraduate degree | Reference | | |
|  | **Obesity** | ------------------------------------- | 0.067 | 1.030 | 0.998-1.064 |
|  | **Income** | Low | 0.741 | 1.106 | 0.609-2.009 |
|  |  | Middle | 0.110 | 1.506 | 0.911-2.487 |
|  |  | High | Reference | | |
|  | **Job Sector** | Government | 0.465 | 1.144 | 0.797-1.644 |
|  |  | Private | 0.653 | 1.093 | 0.741-1.613 |
|  |  | Unemployed/retired | Reference | | |
|  | | | | | |
| **Waterpipe Smoking** | **Response: Increase** | | | | |
|  |  |  | **P-value** | **OR** | **95% CI** |
|  | **Gender** | Male | 0.023 | 1.636 | 1.070-2.501 |
|  |  | Female | Reference | | |
|  | **Age** | ------------------------------------- | 0.084 | 0.982 | 0.961-1.002 |
|  | **Education** | High school and less | 0.023 | 2.473 | 1.136-5.384 |
|  |  | Community college diploma | 0.224 | 1.706 | 0.721-4.038 |
|  |  | Bachelor’s degree | 0.062 | 1.998 | 0.966-4.132 |
|  |  | Postgraduate degree | Reference | | |
|  | **Obesity** | ------------------------------------- | 0.214 | 0.973 | 0.932-1.016 |
|  | **Income** | Low | 0.061 | 3.452 | 0.945-12.600 |
|  |  | Middle | 0.048 | 3.492 | 1.013-12.042 |
|  |  | High | Reference | | |
|  | **Job Sector** | Government | 0.278 | 1.321 | 0.799-2.185 |
|  |  | Private | 0.180 | 1.455 | 0.841-2.520 |
|  |  | Unemployed/retired | Reference | | |
|  | **Response: Decrease** | | | | |
|  | **Gender** | Male | 0.682 | 0.926 | 0.643-1.335 |
|  |  | Female | Reference | | |
|  | **Age** | ------------------------------------- | 0.776 | 1.002 | 0.986-1.019 |
|  | **Education** | High school and less | 0.696 | 1.126 | 0.621-2.043 |
|  |  | Community college diploma | 0.610 | 1.181 | 0.623-2.240 |
|  |  | Bachelor’s degree | 0.428 | 1.232 | 0.735-2.065 |
|  |  | Postgraduate degree | Reference | | |
|  | **Obesity** | ------------------------------------- | 0.914 | 1.002 | 0.968-1.037 |
|  | **Income** | Low | 0.284 | 0.664 | 0.314-1.404 |
|  |  | Middle | 0.932 | 0.972 | 0.509-1.858 |
|  |  | High | Reference | | |
|  | **Job Sector** | Government | 0.294 | 1.248 | 0.825-1.889 |
|  |  | Private | 0.758 | 1.075 | 0.677-1.708 |
|  |  | Unemployed/retired | Reference | | |
|  | **Response: No change** | | | | |
|  | **Gender** | Male | 0.000 | 0.509 | 0.378-0.687 |
|  |  | Female | Reference | | |
|  | **Age** | ------------------------------------- | 0.019 | 1.016 | 1.003-1.030 |
|  | **Education** | High school and less | 0.001 | 0.466 | 0.292-0.743 |
|  |  | Community college diploma | 0.172 | 0.709 | 0.433-1.161 |
|  |  | Bachelor’s degree | 0.141 | 0.746 | 0.505-1.102 |
|  |  | Postgraduate degree | Reference | | |
|  | **Obesity** | ------------------------------------- | 0.870 | 1.002 | 0.975-1.031 |
|  | **Income** | Low | 0.071 | 0.580 | 0.321-1.047 |
|  |  | Middle | 0.559 | 0.858 | 0.515-1.432 |
|  |  | High | Reference | | |
|  | **Job Sector** | Government | 0.076 | 1.350 | 0.969-1.881 |
|  |  | Private | 0.517 | 1.130 | 0.781-1.633 |
|  |  | Unemployed/retired | Reference | | |
|  | | | | | |
| **E-Cigarettes** | **Response: Increase** | | | | |
|  |  |  | **P-value** | **OR** | **95% CI** |
|  | **Gender** | Male | 0.972 | 0.989 | 0.524-1.865 |
|  |  | Female | Reference | | |
|  | **Age** | ------------------------------------- | 0.034 | 0.967 | 0.937-.998 |
|  | **Education** | High school and less | 0.175 | 2.216 | 0.701-7.006 |
|  |  | Community college diploma | 0.302 | 1.953 | 0.547-6.966 |
|  |  | Bachelor’s degree | 0.224 | 1.932 | 0.669-5.579 |
|  |  | Postgraduate degree | Reference | | |
|  | **Obesity** | ------------------------------------- | 0.286 | 0.965 | 0.905-1.030 |
|  | **Income** | Low | 0.877 | 1.008 | 0.365-2.556 |
|  |  | Middle | 0.323 | 1.643 | 0.632-3.735 |
|  |  | High | Reference | | |
|  | **Job Sector** | Government | 0.074 | 2.179 | 0.927-5.125 |
|  |  | Private | 0.005 | 3.670 | 1.474-9.139 |
|  |  | Unemployed/retired | Reference | | |
|  | **Response: Decrease** | | | | |
|  | **Gender** | Male | 0.986 | 1.000 | 0.949-1.052 |
|  |  | Female | Reference | | |
|  | **Age** | ------------------------------------- | 0.193 | 0.984 | 0.961-1.008 |
|  | **Education** | High school and less | 0.795 | 1.120 | 0.478-2.621 |
|  |  | Community college diploma | 0.992 | 0.995 | 0.372-2.660 |
|  |  | Bachelor’s degree | 0.688 | 1.161 | 0.560-2.407 |
|  |  | Postgraduate degree | Reference | | |
|  | **Obesity** | ------------------------------------- | 0.986 | 1.000 | 0.949-1.052 |
|  | **Income** | Low | 0.987 | 1.008 | 0.383-2.656 |
|  |  | Middle | 0.221 | 1.643 | 0.742-3.638 |
|  |  | High | Reference | | |
|  | **Job Sector** | Government | 0.242 | 1.468 | 0.772-2.791 |
|  |  | Private | 0.111 | 1.761 | 0.878-3.534 |
|  |  | Unemployed/retired | Reference | | |
|  | **Response: No change** | | | | |
|  | **Gender** | Male | 0.000 | 0.455 | 0.314-0.658 |
|  |  | Female | Reference | | |
|  | **Age** | ------------------------------------- | 0.666 | 0.996 | 0.981-1.013 |
|  | **Education** | High school and less | 0.278 | 0.725 | 0.406-1.295 |
|  |  | Community college diploma | 0.841 | 1.069 | 0.556-2.054 |
|  |  | Bachelor’s degree | 0.734 | 0.918 | 0.562-1.500 |
|  |  | Postgraduate degree | Reference | | |
|  | **Obesity** | ------------------------------------- | 0.730 | 0.994 | 0.959-1.029 |
|  | **Income** | Low | 0.144 | 1.600 | 0.852-3.004 |
|  |  | Middle | 0.001 | 2.363 | 1.401-3.985 |
|  |  | High | Reference | | |
|  | **Job Sector** | Government | 0.923 | 0.979 | 0.641-1.495 |
|  |  | Private | 0.376 | 1.238 | 0.771-1.987 |
|  |  | Unemployed/retired | Reference | | |
